# Editorial Chit-Chat

**Published:** 1875-07

**Authors:** 


### Editorial Chit-Chat.

HOLLOWAY'S ASYLUM.—*Punch* furnishes this inscription for the front of the idiot asylum founded by Mr. Holloway, who made his fortune in patent medicines:

"Not oft' is fate so just—see wealth restored
Back to the simple source from which it poured."

VEHICLE FOR MEDICINE.—H. says he knows of a man who was ordered by his family physician to take a pill in some "convenient vehicle," and that he sat down in his wheelbarrow to swallow the dose, giving as a reason that he didn't keep a carriage.

THAT VACATION is the cause for delay in the appearance of the BISTOURY. Not arriving at home from the woods until the day that our journal should have been mailed to our subscribers, and having performed no literary work whatever while we were gone, of course, we are late. We hinted in our April number that we were likely to be tardy this time, so that our readers doubtless guessed the cause of our delay before now.

AFTER A BALL.—The somewhat celebrated Count de Grasse was once wounded in the leg with a musket ball. He was brought to a hospital, where the doctors probed, cut, hacked, and made sundry and various incisions in their vain endeavor to find the ball, while the Count looked cooly on and finally ventured to enquire what they were hacking him up so for. "We are seeking for the ball," said the surgeon. "Why in thunder didn't you mention that before," replied the Count; "I have the ball in my pocket."

FIFTH WARD STEAMER.—That was a magnanimous proposition of Ald. Arnot, and acquiesced in by the Common Council, that the new steam fire engine should be located in the 5th Ward—precisely where it is most needed; *provided* the citizens of the ward would purchase a team for the use of the steamer. We hope no resident of the fifth ward will subscribe a shilling for the carrying out of such a ridiculous proposition. Why should not the other wards who have steamers within their lines, purchase horses also? Why should not the property owners along State street pay for the proposed sewer, upon the same principle? If such a demand is persisted in, we hope the voting population of the fifth ward will turn out en masse upon the occasion of all special elections for the expenditure of money, for public improvements, on the other side of the river, and vote—well, as would be natural under the circumstances.

A MEAN PATIENT.—A doctor of North Guilford, Conn., tried to collect a bill from a patient the other day, when it was offset by a long, itemized bill for dinners, horse-baitings, presents of milk, fruit and vegetables, all of which had been tendered the doctor, ostensibly out of good-will. Juries behave queerly oftentimes, and the present case was no exception, as they forced the doctor to pay $7 to get square with his patient.

A DOUBLE HEADED TROUT.—A Mr. Cole, of Whitesville, N. Y., writes to the *Rural New Yorker*, that he has in his trout-pond, a brook trout with two heads.

Gracious, that's nothing,—"Shorty," at Ralston, can discount that trout. He says—*says*, mind you—that he caught a trout on pleasant stream, last year, that had but one eye, and that, like Cyclops of old, was in the middle of his forehead.—Next!

FATHER STACK.—This gentlemen, who has rendered himself famous by his persistent opposition to the Bishop of Scranton, in what Father Stack considers arbitrary and uncanonial powers, has been delivering a series of lectures, throughout Pennsylvania, which have been largely attended and extensively commented upon by the press.—Father Stack has also contributed a lengthy paper to HARPERS WEEKLY, upon the danger to our public schools from the opposition of the Catholic Bishops. Father Stack is a highly educated gentleman, a vigorous writer and an able speaker, so that his influence will be felt throughout the land.

THE FOURTH.—It has past and gone, to make way for the more pretentious one that all the world will assist in celebrating, at Philadelphia, next year. The boys have fired their last cracker, exploded the last torpedo, and sent on a voyage to the clouds, the last rocket. Fanny, the mare, no longer pricks her ears and prances at the sight of a lighted cigar or a fire-fly,—anticipating a momentary explosion, and the family cat inspects the debris with which the ground is covered from the day's pyrotechnic display, from the top of our neighbor's barn, in seeming indecision whether it is safe to occupy her old quarters on the door step; the children have trudged up stairs, with their arms full of empty roman-candles and spin-wheels, and, as we hear their tired "good night," we light our pipe and settle down to a quiet smoke, blessing our stars that the day with its attending confusion and noise has passed, giving us a rest until we all light a cracker in the grand celebration of next year.

DR. GLEASON'S RETURN.—Dr. and Mrs. Gleason have been traveling in the state of California for the past four months, and have returned to us with glowing accounts of the beautiful scenery and invigorating air of the golden state. The next best thing to making a journey oneself, is to listen to some observing person, capable of imparting to others, in a lively, animated chat, what he himself has seen. This qualification the Doctor possesses in an eminent degree, and we hope the Y. M. C. A. will pursuade him to give us the benefit of his travels in the Yosemite and other points of interest in the west.

---

A STRONG ENQUIRY.—Recently, a young man from a neighboring city, was seen to pass back and forth on Water street anxiously examining the signs in his way, until he encountered a prominent bookseller, when the following dialogue was overheard:

The Stranger: "Say, can you tell me where in h—l I can find Jesse C. ?"

Bookseller: "You will not find him there at all, sir; Jesse is regarded as a very upright and honorable gentleman, in this community, and it is not expected that he will be found in the place to which you refer." Further along, a friend informed the stranger that Jesse could be found in the rolling mill, whither he directed his footsteps, muttering something about not being mistaken about the heat of the place, if he was in the name.

---

FLOOD THE LOW GROUND.—What a splendid place we might have for pleasure boats, now rapidly multiplying upon our river, if a brush dam were thrown across the stream, just below the city. It would not only afford us a place for pleasure, but would flood the low places in the rear of the buildings on Water street, and so hide from sight and smell, the garbage that is allowed there to accumulate. A brush dam could be constructed, and one that would be durable and lasting, for less than two thousand dollars—so we have heard it estimated—and would convert the Chemung into a beautiful sheet of water, where it now passes through our city. Will not our daily papers present the feasibility and desirability for such an enterprise, and induce some one to solicit subscriptions of money to construct the dam?

---

EXERCISE.—We cannot conceive of exercise more enjoyable,—always excepting casting the fly for trout, than to construct flower-beds, and to plant therein the endless variety of beautiful flowers that bloom all summer long, lending their beauty and fragrance to the embellishments of home. The less a fellow knows about floriculture too, the better; because he experiences the extreme felicity of being "blown up" by every female member of the household, while they emphatically assure you that not one of the plants will live in such soil as you have prepared for them. The women must have a flower bed made of muck, sand, leaf-mould, guano, plaster, charcoal, and a Granger only knows what else, before they consider it safe to transplant even a sunflower or hollyhock.

But you should see *our* flower-beds. In shape, they are not orthodox, and therefore are in no danger of ever being worshiped; for they "resemble nothing on earth nor in the sea beneath," but are confoundedly crooked and very spacious. Notwithstanding, the flowers bloom, and grow, and smell as sweetly and shake their pretty heads as saucily as though they had been more tenderly cared for by the natural flower-growers—the women. Then, you should see our ferns, taken from the woods in July, carried sixty miles by rail, and transplanted in an unprepared soil, in the sunlight of the city. Ferns! Elegant ones, as graceful, while they tremble in the evening breeze as the plume of a lordly knight, and as tall as the fence against which they are growing. Grow! Of course they grow! notwithstanding the "pure and unadulterated gravel" in which their roots are said to be resting.

To render the exercise exhilerating and sprightly, visit your wife's pet flower garden and extract therefrom her choicest verbenias, a geranium or two, and a few carnations; and if you can manage to step upon and obliterate a few other varieties of plants in the operation, which you do not recognize, and therefore don't care a fig about, why, so much the better, for it will tax your agility in leaping the garden fence with your selections just as your wife appears upon the scene, takes in the situation at a glance, and your coat tails as they disappear over the fence. We have tried it, and can truthfully recommend all but the oration that follows:—don't stop to listen to it—it will not interest you, and you should hasten to deposit your selections in your own beds, where they will be sure to flourish.

For real, delightful exercise, make flower-beds and plant flowers;—all sorts of flowers—at any time of year—at any time of day—in any kind of earth! They'll grow, if you don't pay a particle of attention to what any woman or professional flower-grower directs you to do. If you *do* follow their instructions, your labor is lost, your exercise a failure, and your flowers blasted. Plant on your "own hook" and be happy.

